# Hybrid Drugs—A Strategy for Overcoming Anticancer Drug Resistance?

**DOI:** 10.3390/molecules26092601

**Published:** 2021-04-29

**Authors:** Marta Szumilak, Anna Wiktorowska-Owczarek, Andrzej Stanczak

**Affiliations:** 1 Department of Hospital Pharmacy, Faculty of Pharmacy, Medical University of Lodz, 1 Muszynskiego Street, 90-151 Lodz, Poland; 2 Department of Pharmacology and Toxicology, Medical University of Lodz, Zeligowskiego 7/9, 90-752 Lodz, Poland; anna.wiktorowska-owczarek@umed.lodz.pl; 3 Department of Community Pharmacy, Faculty of Pharmacy, Medical University of Lodz, 1 Muszynskiego Street, 90-151 Lodz, Poland; andrzej.stanczak@umed.lodz.pl

**Keywords:** hybrid drug, conjugate, cancer, drug resistance, overcoming anticancer drug resistance

## Abstract

Despite enormous progress in the treatment of many malignancies, the development of cancer resistance is still an important reason for cancer chemotherapy failure. Increasing knowledge of cancers’ molecular complexity and mechanisms of their resistance to anticancer drugs, as well as extensive clinical experience, indicate that an effective fight against cancer requires a multidimensional approach. Multi-target chemotherapy may be achieved using drugs combination, co-delivery of medicines, or designing hybrid drugs. Hybrid drugs simultaneously targeting many points of signaling networks and various structures within a cancer cell have been extensively explored in recent years. The single hybrid agent can modulate multiple targets involved in cancer cell proliferation, possesses a simpler pharmacokinetic profile to reduce the possibility of drug interactions occurrence, and facilitates the process of drug development. Moreover, a single medication is expected to enhance patient compliance due to a less complicated treatment regimen, as well as a diminished number of adverse reactions and toxicity in comparison to a combination of drugs. As a consequence, many efforts have been made to design hybrid molecules of different chemical structures and functions as a means to circumvent drug resistance. The enormous number of studies in this field encouraged us to review the available literature and present selected research results highlighting the possible role of hybrid drugs in overcoming cancer drug resistance.

## 1. Introduction

Despite enormous advances in the field of cancer therapy that enable the targeting of particular metabolic and signaling pathways or structures within a cancer cell, malignancies still have their mysteries and can outsmart drugs. Resistance to anticancer drugs may be intrinsic and exists before their administration, or arises in response to treatment e.g., due to dosing mistakes, bioavailability changes, or biotransformation fluctuations [1]. The mechanisms for developing cancer resistance include excessive drug efflux or reduced drug uptake, drug inactivation, target alterations, epigenetics, DNA damage repair, apoptotic pathway blocking, epithelial-mesenchymal transition (EMT), tumor microenvironment, and heterogeneity [2,3,4,5]. In addition, the manner of developing acquired resistance can be different in response to standard antineoplastic drugs and targeted therapy [6]. Resistance to cytotoxic drugs usually emerges due to overexpression of ABC transporters in cells membrane, increased activity of detoxification enzymes, or dysregulation of mechanisms involved in DNA homeostasis. In case of targeted therapy, resistance usually develops because cancer cells figure out how to bypass specific blockades in signaling pathways involved in proliferation, survival and cell death. As a consequence, the obstacles preventing continuous growth and cell division are removed, and cancer expansively attacks the host organism [6]. The aforementioned mechanisms of drug resistance can act independently or in combination. For example, acquired taxane resistance is considered to be mediated by altered intracellular drug levels, tubulin structure variations, as well as dysregulation of signal transduction or apoptotic pathways [7]. The coexistence of several resistance mechanisms further complicates responses to treatment [8].

Increasing knowledge of the molecular complexity of cancers and mechanisms of their resistance to anticancer drugs, as well as extensive clinical experience, indicate that it is nearly impossible to achieve desirable chemotherapeutic effects in the treatment of advanced cancer using single drug therapy [9]. Multi-target approach provides not only greater therapeutic anticancer benefits and improved safety, but also reduces the risk of developing drug resistance [6]. The concept of polypharmacology was proposed by Andrew L. Hopkins, who emphasized a marked shift in drug design philosophy from “the one gene-one drug-one disease” paradigm to the “network-targeted-multi-component therapeutics” paradigm. Multi-targeted therapy may be achieved by multi-drug combination cocktails, multicomponent formulations, or multi-target drugs [10].

The superiority of combination therapy over monotherapy was noticed in 1960s, when pediatric patients with acute lymphocytic leukemia were treated with the combination of methotrexate, 6-mercaptopurine, vincristine, and prednisone (formally known as the POMP regimen) [11]. Consequently, during the 1960s and 1970s, many new combined therapeutic protocols using several chemotherapeutic drugs with different mechanisms of action were developed [12,13]. Examples include the MOPP regimen (cyclophosphamide, vincristine, methotrexate, and prednisone in full doses) as the first combined treatment of Hodgkin’s lymphoma [14,15,16], the ABVD regimen (adriamycin, bleomycin, vinblastine, dacarbazine), which could improve long-term treatment outcomes compared to MOPP, with better tolerance and less severe side effects [17,18], or triple CMF and quadruple CMFP drug regimens (cyclophosphamide, methotrexate, 5-fluorouracil, without/with prednisone) in advanced breast cancer, and which achieved impressive therapeutic results [12,19,20,21,22,23,24]. These first studies on the efficacy of combination therapy opened the way to the development of various therapeutic regimens applicable to the treatment of many neoplastic diseases. Using drug cocktails combining two or more therapeutic agents with different modes of action and non-overlapping toxicities has been a common practice in the clinical management of cancer until today [12].

Although using a combination of drugs is obviously a gold standard of care for many cancer types, a careful literature review reveals the need for continuous improvement of this concept. One of the main obstacles to overcome is differences in pharmacokinetics of drugs given in specific regimens, leading to suboptimal drug concentrations, as well as changed drug ratios at the tumor site. Drugs within combinations are usually given in maximum-tolerated doses, which means that it is not the efficacy at the site of action but, rather, overall toxicity that determines the dose. Pharmacokinetic and pharmacological interactions, sensitive to both dosing and sequencing schedules, are then not taken into account, which usually leads to a lack of drug ratios control at the site of action, poorer therapeutic response, and the development of cancer resistance [25]. The answer to this problem may be combinatorial formulations—co-delivering various drugs which act additively or synergistically [25,26,27,28]. In case of malignant diseases, the process of co-formulation is far more sophisticated than the simple mixing of two active substances in one pill. It is the controlled delivery of several agents based on advances in nanotechnology [29]. Nanoparticulate delivery systems, e.g., liposomes, polymeric micelles polymer-drug conjugates, allow for more specific drug targeting and delivery by altering the physicochemical and pharmacokinetic/pharmacodynamic properties of the original drugs [30]. They improve biocompatibility and efficacy of drugs incorporated, and reduce their adverse effects [28,31]. In addition, co-encapsulation enables unfluctuating and concurrent delivery of drug combinations, preserving the integration of different drugs’ pharmacokinetics by precise synergistic drug ratios [32,33] or controlling sequenced drug release [34]. Many interesting solutions in this field are described in literature [29,35,36,37,38,39,40,41], e.g., Vyxeos^®^, the first in a new class of liposomes approved in 2017 by the FDA and in 2018 by the EMA, enables the simultaneous delivery of two drugs, cytarabine and daunorubicin, in a fixed 5:1 molar ratio to increase treatment efficacy of acute myeloid leukemia (AML) with a lower cumulative dose [42]. The major challenge of aforementioned strategies is that safety and efficacy of drugs have to be proven for each individual drug, as well as for their combination. Therefore, combinations of approved drugs are usually evaluated [10,30]. It seems that, in regard to new drug registration, the approval of a single molecule with multiple activities would be far easier in terms of pharmacokinetics and bioavailability issues [10]. Taking this into account, together with obvious fact that an effective anticancer therapy should consider the biological system as a whole instead of a single pharmacological target, designing hybrid drugs which simultaneously target many points of cancer signaling networks has become a popular approach when searching for new effective drugs which are able to overcome acquired cancer resistance [43]. These agents, termed “single molecule multiple targets”, “multiple ligands”, or “hybrid” anticancer agents, have attracted much attention in recent years. Published reviews described the topic from many points of view [44,45,46,47]. Due to enormous amount of research available in this field, we cannot provide the exhaustive data of all hybrid compounds designed to improve cancer treatment effectiveness. Thus, we decided to review the available literature and present selected research results, highlighting the possible role of hybrid drugs in overcoming cancer drug resistance. We searched Medline, Embase, Web of Science Core Collection, and The US National Institutes of Health Ongoing Trials Register (ClinicalTrials.gov) to retrieve all relevant references. The keywords used included: hybrid drug, conjugate, cancer, drug resistance, overcoming anticancer drug resistance, and names of investigated compounds were appropriate. No restrictions were placed on the date of publication when searching the electronic databases.

## 2. Hybrid Drug—What Could It Be?

Hybrid drugs, also termed “single molecule multiple targets” or “multiple ligands” [44], can be referred to as the most sophisticated form of combination therapy. They are designed utilizing molecular hybridization—a strategy of rational drug design which enables the fusing of one or more bioactive compounds or their pharmacophoric subunits into one molecule, which represents the preselected, desired features of original drugs [48]. Obviously, connected entities should retain affinity to their specific targets and provide a superior therapeutic effect by amplification or exerting multifactorial biological activity [26]. Such a single hybrid agent can modulate multiple targets involved in proliferation and efficiently destroy cancer cells. In addition, it possesses simpler pharmacokinetic profile, which reduces drug–drug interactions and facilitates the process of drug development. Moreover, a single medication is expected to enhance patient compliance due to a less complicated treatment regimen, as well as diminished number of adverse reactions and toxicity [45,49]. The concept of hybrid molecules should not be confused with the concept of pro-drugs, since the latter are designed to correct pharmacokinetic and pharmacodynamic profiles of the leading entity [50].

Multi-targeted agents are usually designed according to a multi-pharmacophore mode. IUPAC defines pharmacophore as “an ensemble of steric and electronic features that is necessary to ensure the optimal supramolecular interactions with a specific biological target and to trigger (or block) its biological response” [51]. The multi-pharmacophore model can be divided into four distinct types, according to the degree of pharmacophores overlapping: conjugated pharmacophore (with non-cleavable or cleavable linker), fused-pharmacophore, and merged-pharmacophore [47,52].

Pharmacophores, or whole parent compounds, can be connected by non-hydrolysable, or enzymatically stable spacers, as well as cleavable linkers such as esters, amides, carbamates, or disulfide bonds. The release of parent molecular structures occurs in the particular environment of a targeted tissue. In case of fused hybrids, subunits are in direct contact through a functional group of each molecule, whereas merged hybrid compounds are obtained by overlapping/combining two drug pharmacophores, generating a novel, smaller chemical entity with saved pharmacological properties but substantially different chemical structure from the original drugs or pharmacophores [52,53,54]. Pharmacophores can be derived from drugs acting through the same mechanism of action or through different mechanisms of action [46].

The work on obtaining an efficient hybrid drug is a complex, multi-stage process based on a thorough understanding of targets and chemical properties of their ligands [49]. First, it demands the discovery of multi-target synergy, which is usually inspired by a clinically effective combination of drugs. Next, the key pharmacophores of both active molecules and their appropriate linkage are identified. Research on the structure–activity relationship (SAR) and optimization of a molecular structure, enabling a selection of lead compound for further studies, are extremely difficult tasks [55]. Obtaining merged hybrids demands fusing pharmacophores into a single molecule which can affect the affinity and selectivity profiles of parent compounds, or lead to unintended interactions with additional targets. In case of hybrid molecules, with subunits tethered by non-cleavable linkers, simultaneous interaction with both targets may be impaired because it is difficult to combine two different entities while maintaining their biological properties [56]. The chemical stability of cleavable hybrids is another concern. An appropriate linker should be used to ensure a controlled, tumor-specific cleavage reaction, releasing active drugs at the site of action, with sufficient stability in the bloodstream to avoid systemic toxicity [54]. It is also a challenge to properly adjust the ratio of parent compounds and achieve an adequate modulation of both targets in vivo at similar plasma concentrations [26]. Moreover, it must be underlined that hybrid drugs, as large and complex chemical entities, usually have high molecular mass and high lipophilicity. They violate Lipinski’s [57] and Veber’s [58] rules defining molecular properties influencing oral bioavailability. Therefore, an optimization of drug-like properties of hybrid compounds can be problematic [54,56]. Sometimes, despite many efforts, the potency of the hybrid drug is lower than its primary molecules [59].

## 3. Hybrid Drugs as an Answer to the Anticancer Drug Resistance Problem?

Since scientific facts and clinical practice indicate that multifactorial diseases such as cancer can be effectively treated by mainly attacking multiple targets, hybrid drugs seem to be the best solution to avoid or combat cancer resistance which leads to therapeutic failures. Hybrid molecules are defined as chemical entities with two or more structural domains having different biological functions [60]. As a consequence, designing hybrid drugs which are able to interact with different targets responsible for the onset of cancer offers a unique opportunity to enclose a combination of drugs in one molecule which exhibits increased therapeutic efficacy, reduced toxicity, and which is less vulnerable to developing cancer resistance [61].

Moreover, the strategy of molecular hybridization, with the expanding knowledge about mechanisms of tumorigenesis and cancer resistance, is a potent tool for overcoming cancer drug resistance based on mechanistic rationale. Using properly designed hybrids may ensure the affecting of different hallmarks of cancer simultaneously to avoid upregulation of resistance mechanisms. In addition, a lot of research describes the rational design of hybrid drugs, which can circumvent the already existing resistance of cancer cells. The most common resistance mechanisms and hybrid drugs rationally designed in response to their emergence are briefly discussed below.

### 3.1. Overcoming Anticancer Drug Resistance—From the Drug Perspective

Simultaneous modulation of additional biological targets, or synergistic/additive activity against the same target, can be beneficial for treating malignancies that develop resistance to a particular group of drugs. Cisplatin and platinum(II)-based analogs are approved for the treatment of many solid tumors [62,63,64]. Unfortunately, these DNA-damaging drugs exhibit severe toxicity due to a lack of selectivity. Moreover, therapy often becomes inefficient because drug resistance occurs due to detoxification, decreased cellular uptake, improved DNA repair, increased drug efflux, and diminished apoptosis [65,66].

Many hybrid drugs were proposed as an option to circumvent the limitations of Pt(II)-based chemotherapy [67]. Improved physicochemical, pharmacological, and toxicological properties of Pt(IV) prodrugs containing active ligands in the axial position to the cisplatin molecule were reported [66,67,68]. The insertion of one or two biologically active ligands within the platinum(IV) scaffold produced many dual acting Pt(IV) hybrids. While the Pt(IV) complex undergoes intracellular reduction to produce active Pt(II) species, it also releases two biologically active ligands exhibiting specific modes of action, e.g., histone deacetylase (HDAC) inhibition, antitubulin activity, and DNA damaging properties [67].

**Figure 1:**
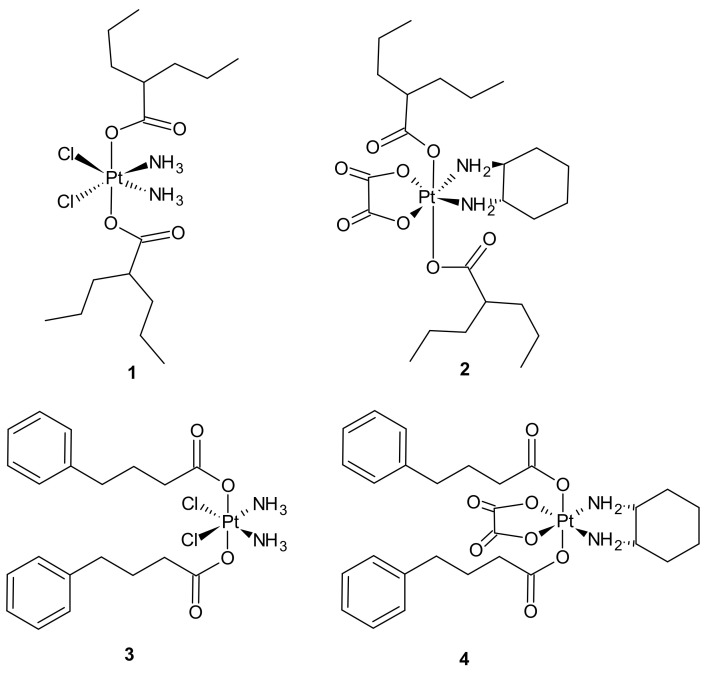
Selected platinum(IV) complexes with valproate (**1**, **2**) or 4-PhB (**3**, **4**) axial ligands.

Pt(IV) derivatives of cisplatin with axial valproate (VPA) ligands **1**, **2** (Figure 1) exhibited enhanced cytotoxicity in cisplatin-sensitive and cisplatin-resistant ovarian cancer cell lines as a result of several processes involving enhanced cellular accumulation, mainly attributed to increased hydrophobicity, downregulation of HDACs, heterochromatin decondensation, and more efficient DNA platination [69].

Further studies were aimed at the explanation of how different platinum moieties (cisplatin vs. oxaliplatin) and histone deacetylase inhibitor (HDACi) axial ligands (VPA vs. phenylbutyrate (PhB)) affect various parameters associated with cytotoxic activity. It was elucidated that ligation of VPA or PhB to the axial positions of the Pt(IV) complex converted them to neutral metalloesters, thereby facilitating a significant intracellular accumulation of both cisplatin and free VPA or PhB. The Pt(IV) derivatives of cisplatin with two VPA **1** or PhB **3** (Figure 1) were considerably more potent than cisplatin (13- and 50-fold respectively), while the VPA **2** or PhB **4** derivatives of oxaliplatin were only approximately as potent as oxaliplatin. In addition, cisplatin complexes were more effective than oxaliplatin complexes. Moreover, as far as the type of axial ligand was concerned, the bis-PhB derivative **3** was approximately 5-fold more potent than bis-VPA analog **1**. Impressive cytotoxicity displayed by the Pt(IV) derivatives of cisplatin with axial PhB or VPA ligands was a consequence of combined intracellular actions of cisplatin and PhB, or VPA that included HDAC inhibition, triggering apoptosis and depleting mitochondrial membrane potential (which precedes mitochondrial-driven apoptotic cell death) [70].

**Figure 2:**
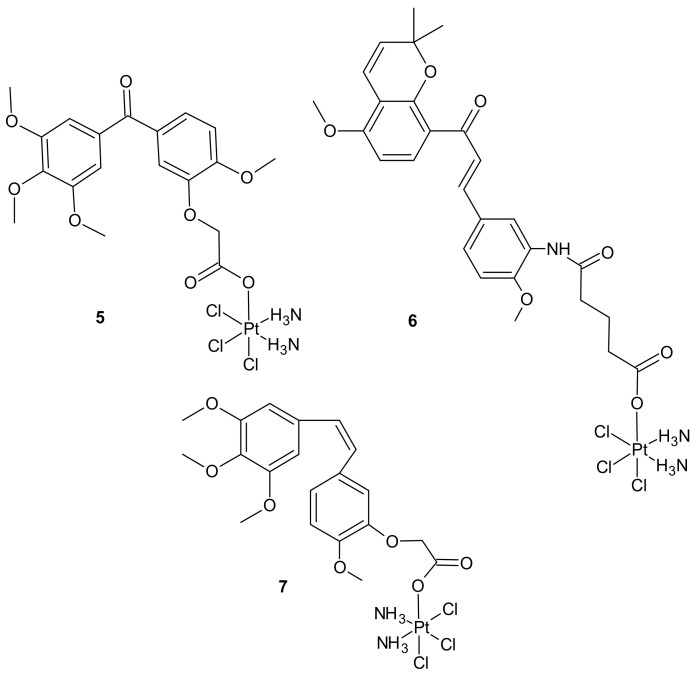
Selected Pt(IV) conjugates with inhibitors of tubulin polymerization.

Pt(IV) conjugates with inhibitors of tubulin polymerization (phenstatin, millepachine, and combrestatin-4 (CA-4) analogs) were also elucidated. Pt(IV) complexes containing a phenstatin analog possessed better antitumor activities than their Pt(II) counterparts against the tested cancer cell lines, including cisplatin-resistant ones, and were less toxic than the corresponding Pt(II) complexes against two normal human cell lines. Further mechanistic evaluation on the most potent complex **5** (Figure 2) revealed that it strongly inhibited tubulin polymerization, arrested the cell cycle at G2/M phases, and markedly enhanced apoptosis. Complex **5** (Figure 2) displayed potent activity against human ovarian SK-OV-3 and human lung A-549 cancer cell lines that were cisplatin-resistant. In addition, it caused apoptotic cell death of human non-small lung cancer cell line NCI-H460 and a potent inhibitory effect on tumor growth in the NCI-H460 xenograft mouse model in vivo. The study suggested that Pt(IV) anticancer prodrugs, containing a small molecule fragment that can stimulate or inhibit tubulin polymerization, may be a promising approach for multiple-targeted cancer therapies [71]. A series of dual-targeting Pt(IV) conjugates consisting of millepachine analogs, with Pt(IV) complexes derived from cisplatin or oxaliplatin, also exhibited the ability to inhibit tubulin polymerization and induce DNA damage. Among them, compound **6** depicted in Figure 2 possessed excellent antitumor activities against the tested human cancer cell lines (including cisplatin-resistant cells) by arresting the cell cycle at the G2/M phases and inducing cell apoptosis, with low cytotoxicity towards human normal cells. In addition, **6** displayed improved antitumor efficacy in the SK-OV-3 xenograft model in comparison to cisplatin and the corresponding millepachine analog. Another Pt(IV) complex with the CA-4 analog, CA-platin **7** (Figure 2), presented potent cytotoxic activities against human cancer cell lines, including cisplatin-resistant cells. CA-platin **7** significantly inhibited tumor growth in the HepG2 xenograft model in vivo. Unfortunately, it was less effective than cisplatin given to mice as a single agent or in combination with CA-4 [68]. The authors supposed that it was attributed to the inefficient release of CA-4 in the body, due to the strong ether bond in the hybrid molecule preventing the cleavage of the complex [72].

**Figure 3:**
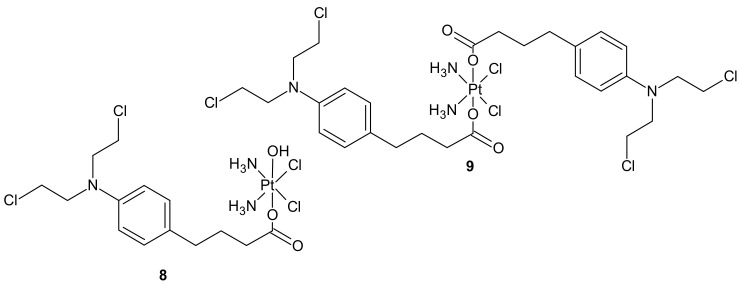
Chlorambucil Pt(IV) conjugates: CLB-Pt **8** and CLB-Pt-CLB **9**.

An interesting strategy for overcoming the resistance of triple-negative breast cancer cell lines (TNBC) to platinum drugs was presented by Ma et al. A performed CompuSyn analysis indicated that cisplatin (Pt) acts synergistically with chlorambucil (CLB) against MDA-MB-231 cells. Two promising platinum(IV) complexes, CLB-Pt **8** and CLB-Pt-CLB **9** (Figure 3), were designed. In vitro, CLB-Pt-CLB **9** (Figure 3) demonstrated better cytotoxicity than cisplatin on MDA-MB-231 cells, apparently due to its dramatically enhanced cellular uptake and DNA platination levels. Compound **9** (Figure 3) efficiently entered cells and released cisplatin and chlorambucil upon reduction. In addition, it enlarged DNA damage and led more cells to apoptosis than cisplatin. It is noteworthy that it exhibited high anticancer activity and no observable toxicity in BALB/c nude mice bearing MDA-MB-231 tumors. The CLB moiety significantly elevated the passive diffusion of **9** (Figure 3) into cells, and damaged DNA synergistically with cisplatin [73].

**Figure 4:**
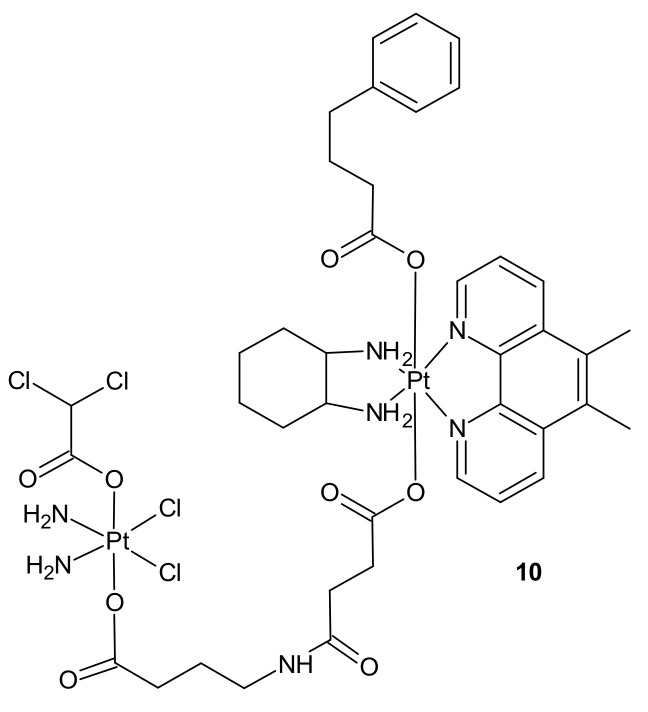
Structure of quadruple action Pt(IV) complex.

Impressive work was performed by Petruzzella and co-workers, who obtained quadruple action Pt(IV) complex **10** (Figure 4), simultaneously releasing four bioactive moieties within cancer cells such as cisplatin, dichloroacetate (DCA), phenylbutyrate (PhB), and Pt56MeSS. Cellular activation of **10** (Figure 4) led to DNA platination, inhibition of HDAC activity caused by PhB, and interference with mitochondrial activity, probably due to the combined actions of Pt56MeSS and DCA. Compound **10** (Figure 4) was extremely potent against highly aggressive KRAS-mutated pancreatic and colon cancer cells [74].

### 3.2. Overcoming Anticancer Drug Resistance—From the Mechanism of Development Perspective

#### 3.2.1. Drug Transporters, Drug Efflux, Drug Uptake

Multidrug resistance (MDR) is referred to as the ability of cancer cells to develop cross-resistance or insensitivity to many anticancer drugs, regardless of their chemical structure, mechanism of action, or molecular target [75]. Drugs affected by multidrug resistance, e.g., vinca alkaloids, anthracyclines, or paclitaxel, are commonly used in a broad spectrum of chemotherapy regimens [5,76,77,78,79]. Although cancer cells develop various mechanisms of resistance to cytotoxic drugs, the key trigger is overexpression of drug transporters from the ABC family in the cell membrane [75]. ATP-binding cassette (ABC) transporters that utilize energy derived from ATP to mediate drug transport are a family of proteins involved in the uptake and efflux of xenobiotics. They play significant roles in the protection of normal cells and tissues by regulating biological membrane permeability [79]. The three major ABC drug transporters—P-glycoprotein (P-gp or ABCB1), Multidrug Resistance Protein 1 (MRP1 or ABCC1), and ABCG2 (also known as MXR-mitoxantrone-resistance gene, BCRP-Breast Cancer Resistant Protein, or Placenta-specific ABC Transporter)—are most frequently associated with unfavorable clinical outcomes through the development of transporter-mediated MDR. They are capable of extruding a wide range of anticancer drugs, thus reducing their intracellular concentration and, subsequently, facilitating the development of resistance [76].

P-glycoprotein (P-gp), a product of the ABCB1 (or MDR1) gene, is profoundly characterized and understood multidrug efflux transporter [80,81]. Since the overexpression of P-gp is one of the major problems hampering successful cancer chemotherapy, many agents have been designed and investigated for their ability to inhibit/modulate P-gp mediated MDR alone, or in combination with cytotoxic agents [82,83]. Nonetheless, the clinical outcomes are still disappointing due to their low potency and specificity. The need to use high doses results in serious side effects and toxicity to normal tissues, owing to the wide distribution of P-gp and its important physiological and pharmacological roles in the human body. The discovery of new and effective P-gp modulators without the abovementioned disadvantages is still a challenging task [84]. Among various means of transient modulation of MDR, such as direct inhibition of ABC transporters, gene silencing, transcriptional regulation, and specific drug-delivery systems [85], hybrid drugs, due to their multiple targeting nature, were also investigated as a promising way of overcoming P-gp-mediated MDR [82].

**Figure 5:**
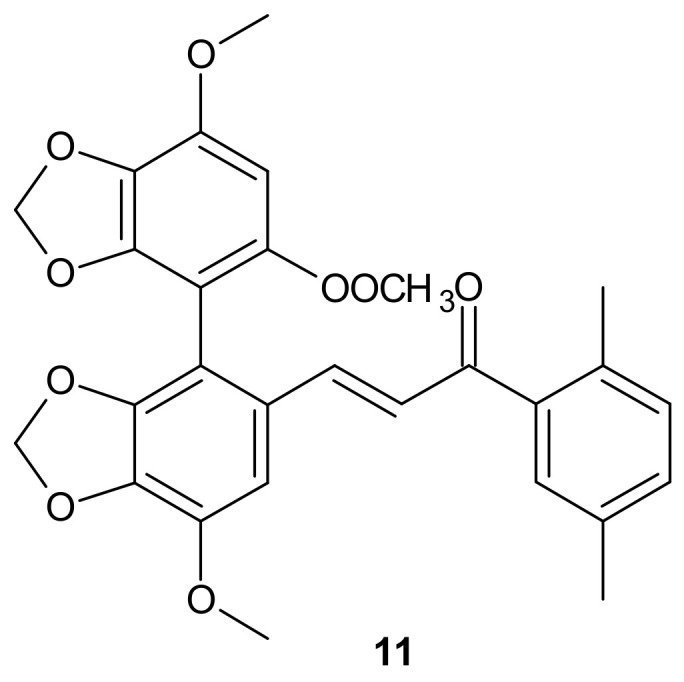
Structure of the most active bifendate-chalcone hybrid against P-gp function.

A series of bifendate–chalcone hybrids were synthesized and evaluated to enhance the P-gp inhibitory effect of bifendate. The most promising compound, **11** (Figure 5), exerted little intrinsic cytotoxicity, whereas its chemosensitizing effect was prolonged and more potent than bifendate and verapamil (VRP). In addition, **11** (Figure 5) did not stimulate P-gp ATPase activity, which suggested that it is not a P-gp substrate [86].

Another concept for overcoming P-gp-associated MDR was the design of hybrid drugs bearing an *N*, *N*-bis(alkanol)amine diester scaffold, combined with a coumarin or benzene sulfonamide moieties, with a synergistic inhibitory activity mechanism on P-gp and carbonic anhydrase [87].

Carbonic anhydrase XII (CA XII) is a membrane enzyme that maintains suitable intra- and extracellular pH in tumor cells. It is highly expressed in some chemoresistant P-gp-positive cancer cells. Since optimal pH for efficient P-gp efflux activity in cancer cells is slightly alkaline, CA XII activity is critical for P-gp efflux. As a consequence, the activity of P-gp can be modulated by CA XII inhibition, producing a significant decrease in P-gp ATPase activity [88,89].

**Figure 6:**
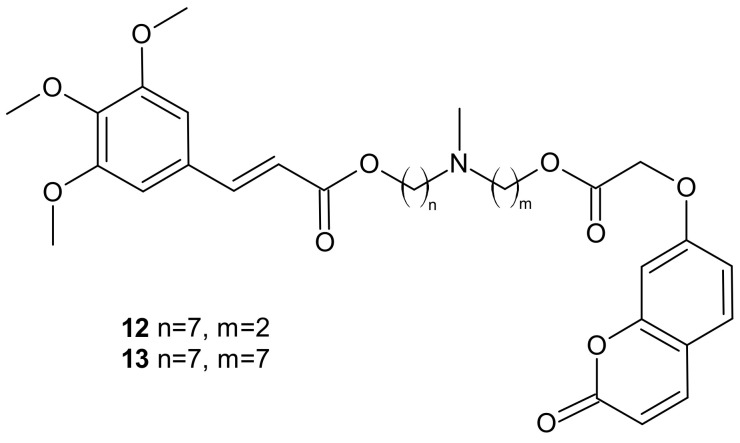
Selected dual P-gp and CA XII inhibitors.

Among the tested compounds, derivatives bearing coumarin scaffolds **12** and **13** (Figure 6), incorporating an (*E*)-3-(3,4,5-trimethoxyphenyl)vinyl moiety linked to a 7-methylenes spacer, showed the best synergistic MDR inhibitory profile, being more potent on LoVo/DOX colon cancer cells that overexpressed both P-gp and CA XII than on K562/DOX leukemia cells overexpressing only P-gp. It made them potentially selective chemosensitizers able to overcome P-gp-mediated MDR with a synergistic antitumor mechanism [87].

**Figure 7:**
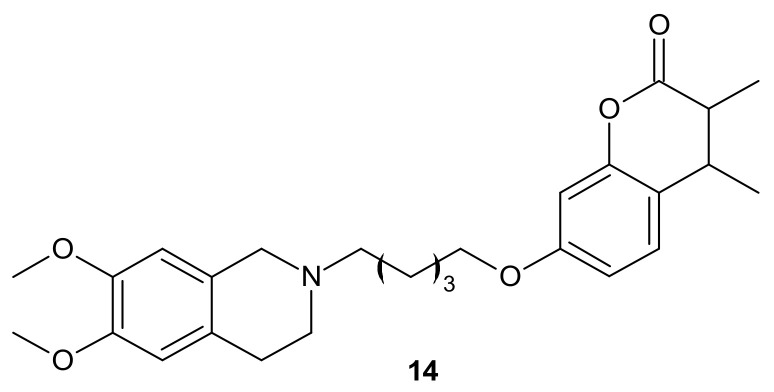
The most potent THIQ-coumarin conjugate.

Rullo et al., obtained conjugates consisting of 1,2,3,4-tetrahydroisoquinoline (THIQ) moiety linked to coumarin scaffold through differently shaped and sized spacers. Compounds were evaluated in Madin-Darby Canine Kidney (MDCK) cells overexpressing P-gp and MRP1. Several THIQ-coumarin conjugates were identified as nanomolar P-gp inhibitors, and the most potent compound, **14**, depicted in Figure 7 with a pentamethylene linker, showed nanomolar inhibition potency. In addition, it was elucidated that linker length and flexibility affected P-gp inhibition potency, whereas bulky groups (Br, Phenyl) at coumarin C3 improved P-gp/MRP1 selectivity. Some novel compounds could reverse the resistance of MDCK-MDR1 cells to doxorubicin [90].

**Figure 8:**
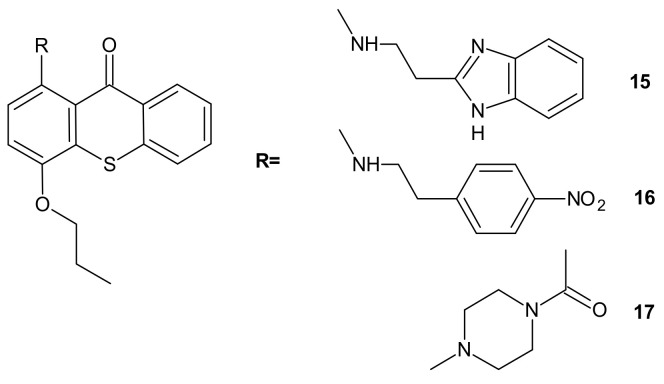
Structures of selected 1-aminated thioxantones.

Hybrid compounds merging a thioxanthonic scaffold and an amine group, described as important pharmacophoric features for P-gp inhibition, were designed using homology modeling and docking by Palmeira et al. [91]. 1-[2-(1*H*-benzimidazol-2-yl)ethanamine]-4-propoxy-9*H*-thioxanthen-9-one **15** (Figure 8) was identified as a potent P-gp inhibitor. It caused an accumulation rate of rhodamine-123 similar to verapamil (a known P-gp inhibitor) in the K562Dox resistant cell line. At a concentration of 10 µM, compound **15** caused a significant decrease in the GI_50_ (growth inhibitory activity) value of doxorubicin in the K562Dox cell line, being 2-fold more potent than verapamil. Compounds **16** and **17** (Figure 8) at 10 µM sensitized resistant K562Dox cells (despite the lower rhodamine-123 accumulation in comparison to verapamil), probably due to their dual activity as P-gp and cell growth inhibitors [91].

**Figure 9:**
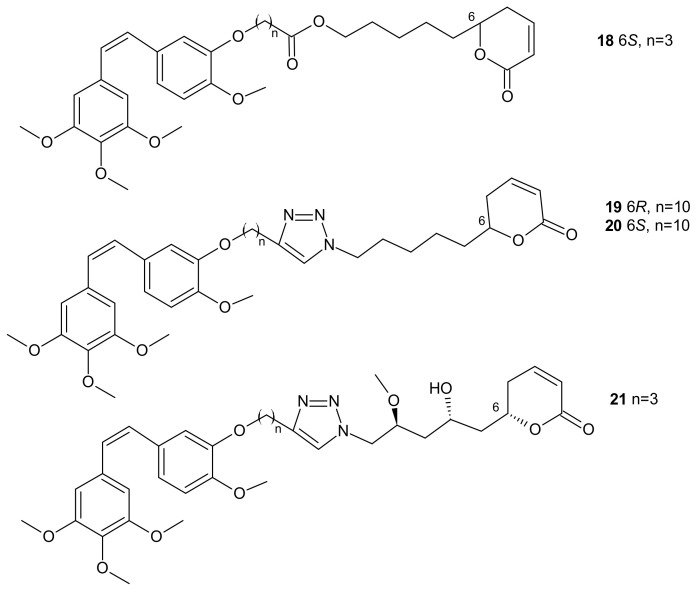
Representative combrestatin A (CA-4) and pironetin hybrid molecules.

In some cases, direct interaction of a hybrid drug with the nuclear target can prevent the excretion of the drug from the cell. Hybrids of combrestatin A (CA-4) and pironetin are a fine example [92]. CA-4 is a plant-derived product which exerts its action by binding to ß-tubulin, at colchicine-binding site [93,94,95], while pironetin (5,6-dihydro-α-pyrone of fungal origin) can bind to α-tubulin [96,97]. Conjugates of CA-4 and pironetin, tethered by an ester-type linker of variable length or a 1,2,3-triazole spacer, were designed to display a dual affinity to both tubulin units. They could bind covalently to tubulin, which prevented the efflux pump from expelling them out of cells. Evaluation against two human ovarian carcinoma cell lines, A2780 (sensitive to chemotherapy) and A2780AD (resistant to chemotherapy), revealed the highest cytotoxicity for three compounds: **18**, **19**, and **20** (Figure 9). The resistance factor (RF—the ratio of the IC_50_ for A2780AD to IC_50_ for A2780) for most of the compounds under study was close to unity, indicating that hybrid molecules of CA-4 and pironetin were also cytotoxic to multidrug resistant A2780AD cells. The effect of hybrid molecules of CA-4 and pironetin on the cell cycle was also evaluated in a non-small-cell lung adenocarcinoma cell line (A-549). Among tested compounds, two conjugates, namely **18** containing ester-type linker and **21** containing triazole linker (Figure 9), exerted the highest antiproliferative activity against A-549 cell line. The same molecules displayed the strongest effect on depolymerization of the microtubule network [92].

Resistance related to drug efflux is common but, sometimes, a lack of therapeutic activity may be associated with impaired carrier-mediated transport of specific drugs into the cancer cell [98].

**Figure 10:**
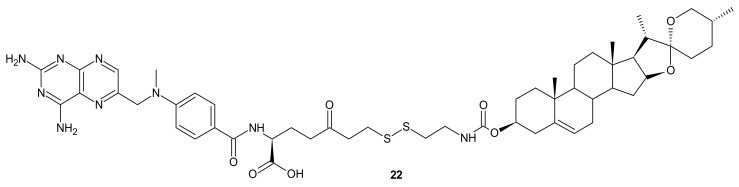
The most potent MTX-diosgenin conjugate.

A deficiency in cancer of a reduced folate carrier mediated transport is the primary and frequent mechanism of resistance to methotrexate (MTX), which limits its clinical use as an anticancer drug [99]. To overcome folate transporter-related resistance, a series of methotrexate-diosgenin conjugates with various polyamine linkers was designed [100]. Diosgenin is a natural, strongly hydrophobic steroidal saponin, with a cholesterol-like structure exhibiting high biocompatibility and anticancer activity [101]. MTX-diosgenin conjugates exhibited a stronger antiproliferative activity against transport-resistant breast cancer cell line MDA-MB-321 than MTX. They had the capability of entering MTX-resistant cells and retained the ability to inhibit dihydrofolate reductase (DHFR), despite diosgenin substitution. Conjugate **22** (Figure 10) possessing disulfide bond was the most active among obtained compounds [100].

**Figure 11:**
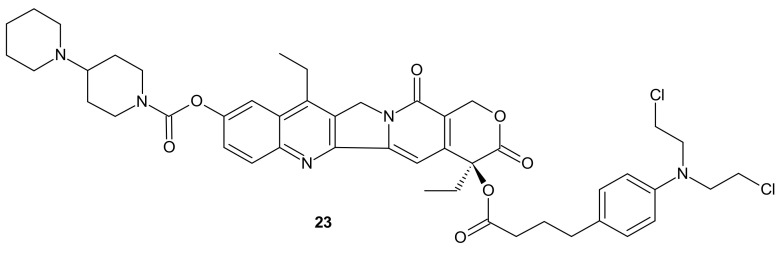
Amphiphilic drug−drug conjugate (ADDC) Ir-Cb, self-assembling into ADDC nanoparticles.

Designing drug delivery systems is often reported as an efficient approach to overcome the efflux phenomena of anticancer drugs [102], but the use of polymeric drug carriers is sometimes associated with a number of obstacles, such as low drug loading, side effects connected with degradation, metabolism, and excretion, as well as poor quality control [103,104]. The carrier-free, drug self-delivery system for cancer therapy proposed by Huang P. et al., seems to be an innovative solution [105]. An amphiphilic drug–drug conjugate (ADDC) **23** (Figure 11), consisting of the hydrophilic anticancer drug irinotecan (Ir) tethered by a hydrolyzable ester linkage to the hydrophobic anticancer drug chlorambucil (Cb), exhibited the ability to assemble into nanoparticles, with longer blood retention half-life in comparison to free parent drugs. It facilitated the accumulation of drugs in tumor tissues and promoted subsequent cellular internalization, helping to avoid P-gp-mediated multidrug resistance. After ADDC **23** (Figure 11) hydrolysis, two released anticancer drugs exerted synergistic cytotoxicity to the tumor cells, exhibiting a higher apoptotic rate and anticancer activity than the individual free drugs. These advantages of Ir−Cb ADDC nanoparticles result in superior anticancer efficacy in vivo [105].

#### 3.2.2. Drug Inactivation (Enzymatic Detoxification)

The glutathione S-transferases (GSTs) represent a family of detoxification enzymes that catalyze the conjugation of glutathione (GSH) to many exogenous and endogenous compounds, including carcinogens, drugs, and oxidative stress products. Several mechanisms, such as transcriptional activation, stabilization of either mRNA or protein, and gene amplification, are reported to be involved in the overexpression of GSTs [106]. MDR related to GSTs overexpression is a consequence of the GSH-anticancer drug conjugates’ formation and their active efflux via ABC transporters, or the inhibition of the mitogen-activated protein kinases pathway (MAPKs) [107]. Many anticancer agents, such as adriamycin, 1,3-bis(2-chloroethyl)-1-nitrosourea (BCNU), busulfan, carmustine, chlorambucil, cisplatin, cyclophosphamide, melphalan, or thiotepa, are potent substrates of GSTs [108,109], which affects their anticancer activity and lead to adverse toxicity effects. [107,110]. Conjugation of platinum-containing cancer drugs, such as cisplatin and oxaliplatin, with GSH leads to their recognition as substrates for ABC transporters and enhances their efflux from the cancer cells [111].

**Figure 12:**
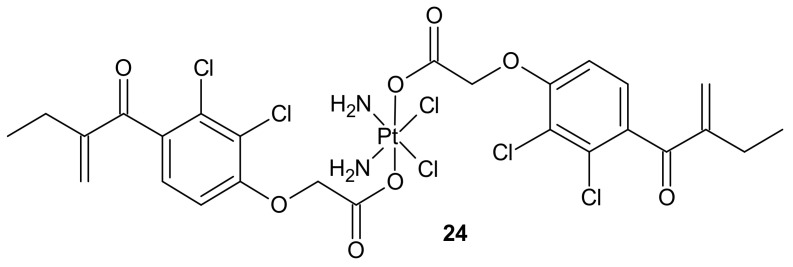
Structure of ethacraplatin (EA-CPT).

To overcome resistance to cisplatin associated with overexpression of π class glutathione S-transferase (GST π-1), ethacraplatin (EA-CPT) **24** (Figure 12), a trans-Pt(IV) carboxylate complex containing ethacrynate ligands, was designed [112]. Metallodrug **24** (Figure 12) strongly inhibited the GST activity in cell-free and cell systems (~10-fold of ethacrynic acid (EA) alone) and accelerated growth inhibition of cisplatin-resistant cancer cells, namely breast MCF-7 and T47D, lung A-549, and colon HT29 human carcinoma cells, in comparison to cisplatin [113]. Unfortunately, in the case of malignant pleural mesothelioma (MPM) cells, compound **24** (Figure 12) could not inhibit cellular GST activity. Therefore, it was postulated that its activity may be related to the type of cancer [114].

#### 3.2.3. DNA Damage Repair

**Figure 13:**
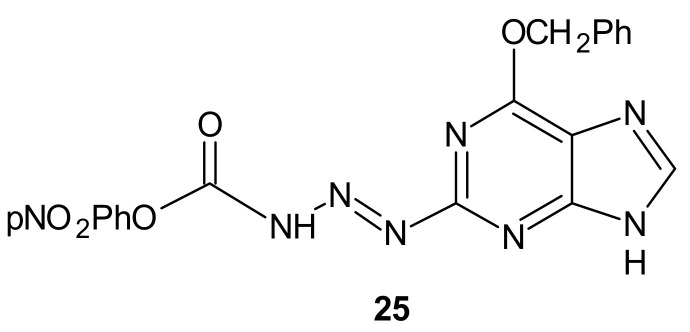
The most potent methyltriazene hybrid bearing O^6^-bnzylguanine moiety.

Many chemotherapeutic drugs, such as methylating, chloroethylating, and platinum-based agents commonly used for treating cancer, exhibit antitumor effects by causing direct DNA damage in tumor cells [115]. Their effectiveness is often negatively influenced by the ability of cancer cells to repair this DNA damage. The role of DNA damage repair in drug resistance was excellently reviewed in [116]. One of the commonly described mechanisms of resistance to alkylating agents is increased O^6^-alkylguanine-DNA alkyltransferase (MGMT) protein activity, which repairs the cytotoxic O^6^-methylguanine (O^6^-BG) DNA adduct through direct transfer of the alkyl group to its cysteine residue [117] and prevents its harmful effects, resulting in cell apoptosis [118]. Since high levels of MGMT in tumor cells result in severe resistance to guanine O^6^-alkylating agents, a series of methyltriazene hybrids bearing DNA methylating triazenes and O^6^-BG was obtained. Hydrolysis of obtained compounds under physiological conditions released O^6^-BG (a pseudosubstrate of MGMT) and DNA-damaging methyldiazonium derivatives, while *p*-nitrophenyloxy derivative **25**, depicted in Figure 13, exhibited favorable penetration properties, and was the most active toward the NCI-60 panel of human tumor cell lines [119].

**Figure 14:**
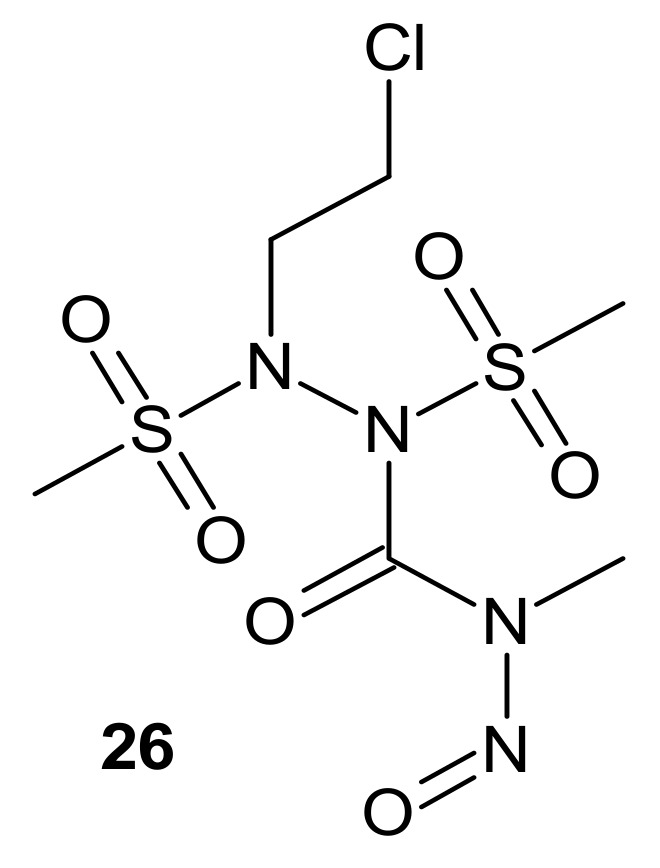
A hybrid compound with dual chloroethylating and methylating functions.

Zhu et al., obtained compounds with dual chloroethylating and methylating functions. Methylating moiety was introduced to deplete MGMT activity, which enhanced the sensitivity of cells to chloroethylating entities, inducing lethal interstrand cross-links. Compound **26** (Figure 14) exhibited the highest toxicity against human prostate cancer cell line DU-145, which expressed MGMT [120].

**Figure 15:**
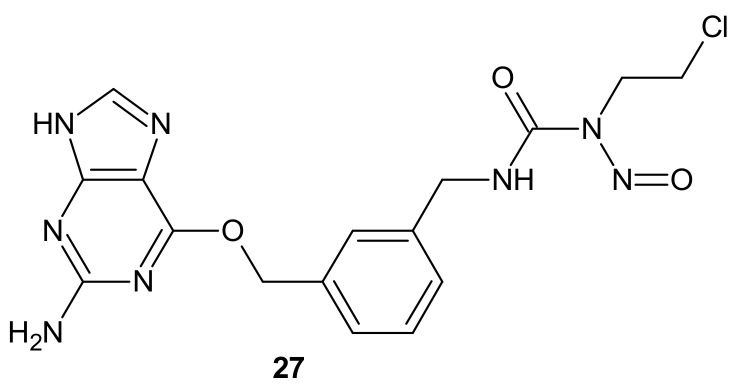
Rrepresentative combi-nitrosourea hybrid.

Combi-nitrosourea molecule **27** (Figure 15), releasing a DNA cross-linking agent and an inhibitor of MGMT, was designed in response to cancer resistance to chloroethylnitrosoureas (CENUs) induced by MGMT, which repairs O^6^-alkylated guanine and, subsequently, inhibits the formation of dG–dC cross-links. It hampers the application of CENUs in chemotherapy regimens. Molecule **27** (Figure 15) exhibited higher cytotoxicity against MGMT high-expressing glioma cells compared with nimustine, carmustine, and their respective combinations with O^6^-BG. The results suggested that the superiority of **27** (Figure 15) is related to the simultaneous release of a chloroethyldiazonium ion, inducing DNA cross-link and O^6^-BG analogs inhibiting the MGMT-mediated drug resistance in MGMT high-expressing cells [121].

**Figure 16:**
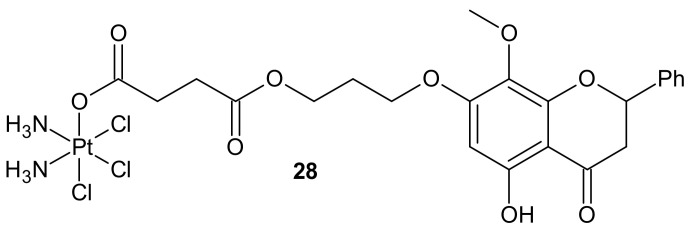
Structure of Pt-wogonin conjugate.

In addition to the previously mentioned hybrid Pt(IV) complexes (see Section 3.1), several platinum hybrids, with bioactive ligands preventing multiple DNA damage response-mediated pathways, were designed [66]. Cis-wogonin **28** (Figure 16) is an octahedral Pt (IV) conjugate containing wogonin (5,7-dihydroxy-8-methoxyflavone, isolated from *Scutellaria baicalensis*, exhibiting multiple anticancer effects on gastric, lung, and glioma cancer cells [122]) as an axial ligand. Cis-wogonin **28** (Figure 16), designed to suppress DDR (DNA damage repair)-related proteins, could reverse existing cisplatin resistance. The significant antitumor activity of Cis-wogonin **28** (Figure 16) was related to its ability to suppress JWA (retinoic acid-induced cytoskeleton-like gene involved in base excision repair (BER) and DNA single-strand break repair (SSB) processes) and its multi-interaction with X-ray repair, cross complementing 1 protein (XRCC1) required for efficient repair of SSBs [123]. Cis-wogonin **28** (Figure 16) seemed to be a promising cytotoxic agent reversing cisplatin resistance by targeting SSBs repair pathways and inducing apoptosis [124].

**Figure 17:**
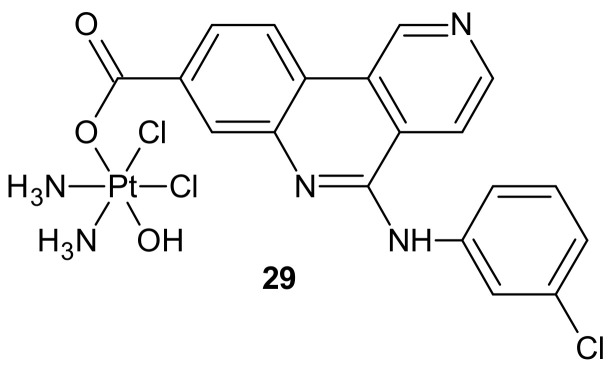
Structure of Cx-platin hybrid.

Another hybrid drug, Cx-platin **29**, showed in Figure 17, was designed to disrupt the DNA damage response mediated by Casein Kinase 2 (CK2). CK 2 is a constitutively active Ser/Thr protein kinase overexpressed in cancer cells. It is involved in regulating many prosurvival cellular processes, playing important roles in resistance to various chemotherapeutics [125,126,127,128,129,130] and the surveillance and repair of both single- and double-strand breaks [131]. Cx-4945 is a selective ATP competitive inhibitor of CK 2, exhibiting activity against numerous malignancies in vitro and in vivo [132,133]. It was elucidated that the antiproliferative activity of Cx-4945 is related to modifications of signaling pathways such as PI3K/AKT, impairing DNA repair response, angiogenesis, splicing regulation, stress-induced cell death, or epigenetic modulation [133,134,135,136,137,138]. The inhibition of CK2 by Cx-4945 strongly enhanced the efficacy of cisplatin [134]. Cx-platin **29** (Figure 17), prepared by a fusion of cisplatin and Cx-4945 as an axial ligand, exhibited superior cytotoxicity in comparison to cisplatin against cancer cell lines, with distinct CK2-expressed levels, through suppressing CK2-mediated DNA damage repair and reversed cisplatin resistance. Due to high lipophilicity and complex stability, **29** (Figure 17) was highly accumulated in tumor cells. Moreover, subsequent profound platination of DNA caused severe DSB and SSB damage, inducing enhanced cell cycle arrest and apoptosis compared with cisplatin and the combination of cisplatin and CX-4945. Further in vivo tests showed that **29** (Figure 17) displayed high tumor inhibition rates and barely any toxicity effects in contrast to cisplatin [139].

#### 3.2.4. Target Modifications

Another important defense line of cancer cells is their ability to camouflage drug targets. Mutation, amplification, as well as over- or under-expression of target levels ultimately lead to drug resistance [1]. Such a mechanism is observed in cases of many anticancer drugs, e.g., tyrosine kinases [140] or antitubulin agents. The latter can be classified as microtubule stabilizers (taxanes and epothilones) that stimulate tubulin polymerization, or destabilizers (vinca alkaloids, colchicine) that inhibit tubulin polymerization [141]. In rapidly proliferating cancer cells, both events lead to cell cycle arrest and apoptosis [142]. Mutations in tubulin subunits, changes in the tubulin isotype composition of microtubules, and alterations in microtubule-regulatory proteins are, apart from P-gp engaged in drug efflux [7,143], main factors influencing antitubulin drug resistance [141]. Growing scientific evidence shows that overexpression of βIII-tubulin is responsible for the taxane resistance appearing in ovarian cancer [144,145,146,147], as well as in other tumor types, such as lung, breast, and gastric cancers [148,149].

It was revealed that compounds binding at colchicine can circumvent β III-tubulin-mediated resistance, which indicates the significant importance of designing new agents targeting colchicine-binding sites in an attempt to overcome taxane resistance in refractory cancers [150].

Zhang and co-workers, inspired by successful combinations of taxanes with receptor tyrosine kinase inhibitors (RTKi), reported on hybrid furo[2,3-*d*]pyrimidines exhibiting potent dual antitubulin and antiangiogenic activities [151].

**Figure 18:**
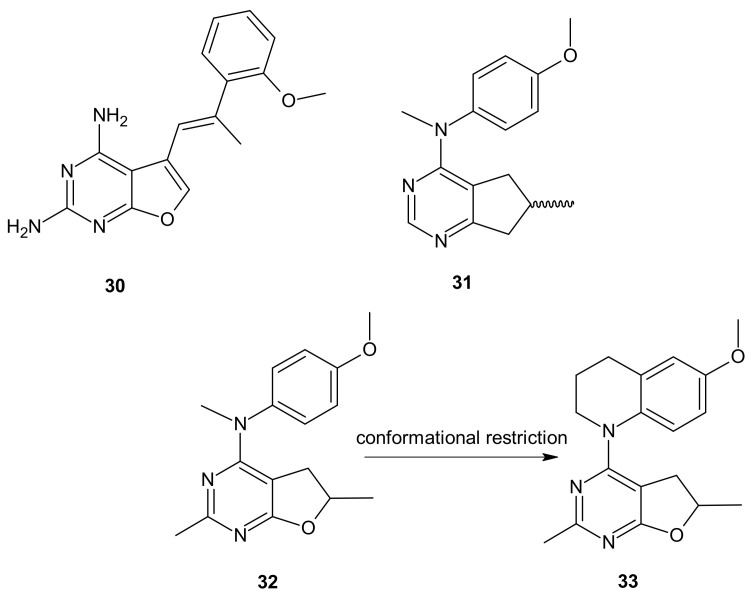
Hybrid furo[2,3-*d*]pyrimidines exhibiting dual antitubulin and antiangiogenic activities.

The choice of pharmacophores was inspired by the results of previous studies reporting that the compound **30** (Figure 18), based on furo[2, 3 − *d*]pyrimidine scaffold, had afforded both VEGFR-2 and PDGFR-β inhibitory activity [152,153], while 6-methyl cyclopenta-fused pyrimidines **31** (Figure 18) exhibited potent tubulin depolymerization activity, together with significant in vitro and in vivo antitumor activity [154,155,156]. Among synthesized derivatives, compound **32** (Figure 18), showed excellent dual RTK (VEGFR-2 and PDGFR-β) and microtubule inhibitory activity. In addition, it exhibited antiproliferative activity against the NCI-60 panel of cancer cells at low nanomolar levels, and was active in either paclitaxel-resistant tumor cell lines with βIII tubulin, or those that overexpressed P-gp. Moreover, **32** (Figure 18) showed potent growth inhibitory activity against tumor cells expressing VEGFR-2 and PDFGR-β, which is comparable to sunitinib, and caused cell cycle arrest in the G2/M phase, with subsequent apoptosis. The microtubule depolymerization through binding at the colchicine site was determined to be the primary mechanism of its antitubulin action. Compound **32** (Figure 18) also provided excellent antitumor activity superior to docetaxel and sunitinib in two murine models, reducing tumor size, vascularity, and metastases without obvious toxicity. An additional advantage of **32** (Figure 18) over clinical antimitotic agents, e.g., paclitaxel, is its simple synthetic procedure and the possibility of its conversion to the water-soluble salt form [151].

The conformational restriction of **32** (Figure 18), leading to the tetrahydroquinoline analog **33** (Figure 18), enabled significant potency improvement against most of the NCI-60 panel cancer cells, including several chemoresistant cell lines. Compound **33** (Figure 18) showed potent RTK inhibition with nanomolar IC_50_ value and exhibited microtubule depolymerizing activity comparable to or better than the lead compound **32** [157].

Deregulation of tyrosine kinases (TK), being essential components of signaling cascades involved in diverse biological processes such as growth, differentiation, metabolism, and apoptosis, is connected with tumor proliferation, invasion, and metastasis, as well as tumor neovascularization. As a consequence, protein kinase inhibitors (TKIs) constitute important molecularly targeted drugs, broadly used for treating various malignancies [158]. Their introduction into clinical practice was a real breakthrough and opened the way to different targeted therapies, significantly improving patient outcomes without the severe toxicity characteristic of commonly used chemotherapeutics [159]. Unfortunately, the usefulness of TKIs is sometimes impaired by poor cancer response and acquired resistance. The predominant mechanism involved in resistance to TKI therapy, apart from gene amplification, overexpression, or alterations of signaling pathways, is the emergence of point mutations of the target kinase at the drug–kinase–interaction domain [140,158,160].

**Figure 19:**
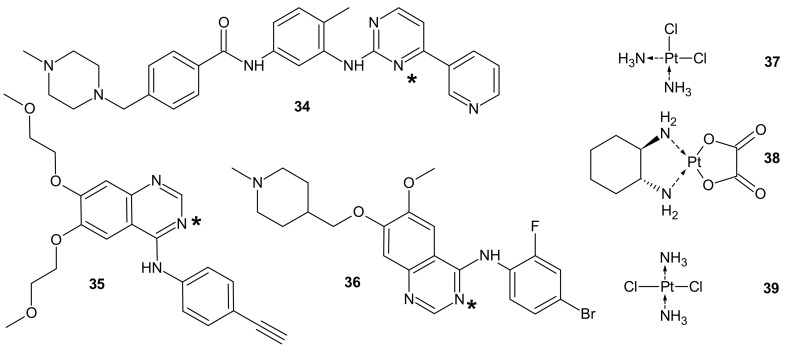
PT-TKI hybrids. Each TKI (**34**–**36**) was conjugated with each platinum derivative (**37**–**39**) through nitrogen atom labelled *.

To circumvent TKI resistance, Pt-TKI hybrid compounds (Figure 19) were obtained by incorporating one of three TKIs (imatinib **34**, erlotinib **35**, and vandetanib **36**) into the core structure of Pt-based anticancer drugs (cisplatin **37**, oxaliplatin **38** and transplatin **39**, Figure 19). Hybrids were formed by the reaction of Pt with the nitrogen atom on the quinazoline ring (erlotinib **35** and vandetanib **36**) and pyrimidine ring (imatinib **34**) of the TKIs. The additional hydrogen bonds facilitating the binding of the hybrids to altered sites made them less prone to resistance, causing secondary mutations. In addition, a dual anticancer mechanism of the Pt-TKI hybrids was observed, encompassing the inhibition of oncogenic kinases and monofunctional platination of DNA, which also probably contributed to the circumvention of drug resistance. The hybrids were not affected by the overexpression of the transporters in resistant cells and might be able to penetrate the blood–brain barrier to treat cancer metastasis in the brain. Apart from being effective in treating tumors bearing both sensitizing and resistance mutations, such as the third-generation mutant selective epidermal growth factor EGFR-TKI, the hybrids are also expected to exhibit a better side effect profile than the first generation TKIs [161].

**Figure 20:**
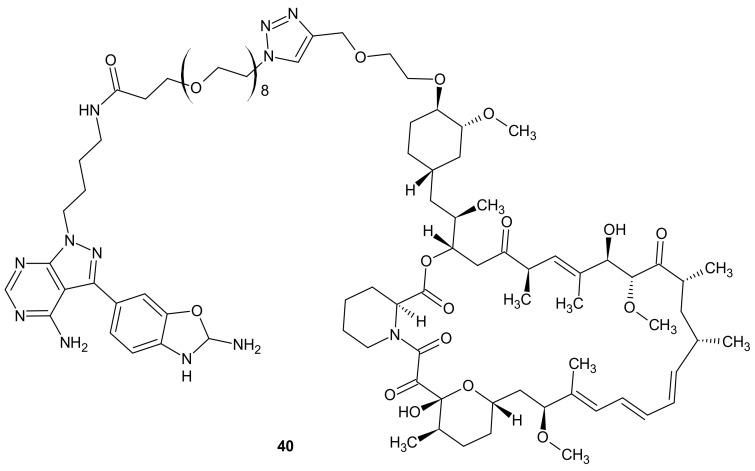
Structure of RapaLink-1.

Mammalian Target Of Rapamycin Kinase (mTOR) mutations, such as mutations located in FRB-domain, as well as mutation located in kinase domain, are responsible for resistance to rapamycin analogs (rapalogs, e.g., temsirolimus, everolimus that are first in class mTOR kinase inhibitors approved for cancer therapy [162]) and second generation mTOR ATP competitive inhibitors (TORKi), respectively [163]. The exploitation of both ATP and FRB binding sites of mTOR generated the third class of mTOR inhibitors, represented by RapaLink-1 **40** and depicted in Figure 20. Compound **40** is a bivalent mTOR inhibitor consisting of rapamycin-FRB binding moiety, appropriately linked to the TORKi molecule. It prevents tumor growth in wild-type mTOR expressing cells and in cells with acquired resistance to the first and the second generation of mTOR inhibitors. A unique bivalent interaction of RapaLink-1 **40** with mTOR is that binding at one site places the second half of the ligand in close proximity for binding to the second site, thus overcoming point mutations that diminish drug binding [163]. In comparison with temsirolimus, which is the first-line therapy of renal cell carcinoma (RCC), **40** (Figure 20) showed significantly improved inhibition of proliferation, migration, invasion, and colony formation in the treatment of sunitinib-sensitive and sunitinib-resistant RCC cells in vitro and in vivo. It suppressed the mTOR signaling pathway, a part of the MAPK signaling pathway, the ErbB signaling pathway, and ABC transporters that are associated with resistance to many drugs [164].

#### 3.2.5. Alterations in Signaling Pathways

Alterations in signaling pathways associated with survival (PI3K, AKT, mTOR, STAT), proliferation (MAPK, STAT, growth factors: FGFs, EGFR, IGFs), and cell death (BCL2 family and PTEN) are resistance mechanisms characteristic of targeted therapies [6].

In case of multifactorial diseases such as cancer, the strong specific inhibition of a particular dysregulated pathway is insufficient to hamper the disease, because changes in signaling networks can reduce the effect of the drug through the activation of new aberrant cellular pathways. Moreover, targeted therapies often unintentionally provoke the preferential growth of resistant cells due to selective pressure on single elements of dysregulated pathways [165].

The PI3K/AKT/mTOR is a common example of a hyper-activated signaling pathway in cancer cells. Phosphatidylinositol 3-Kinase (PI3K) and mTOR synergistically promote tumor progression and resistance to chemotherapy; therefore, inhibition of this pathway is an excellent treatment strategy and an efficient tool to avoid or circumvent cancer chemotherapy resistance [166].

Rapalogs, aforementioned in Section 3.2.4, are partial inhibitors of mTOR through allosteric binding to mTOR complex-1 (mTORC1), but not mTOR complex-2 (mTORC2) [167,168]. Since mTORC1 negatively regulates insulin/IGF-1 receptor signaling, mTORC1 blocking by rapalogs can stimulate PI3K/AKT signaling and antagonize their antitumor efficacy [168,169]. It was elucidated that treatment with rapamycin or rapalogs led to increased PI3K activity and AKT signaling, resulting in minimal inhibition of tumor cell growth [170,171]. Co-targeting mTOR and PI3K/AKT signaling prevented mTOR inhibition-initiated AKT activation and enhanced antitumor effects in vitro and in vivo [172].

**Figure 21:**
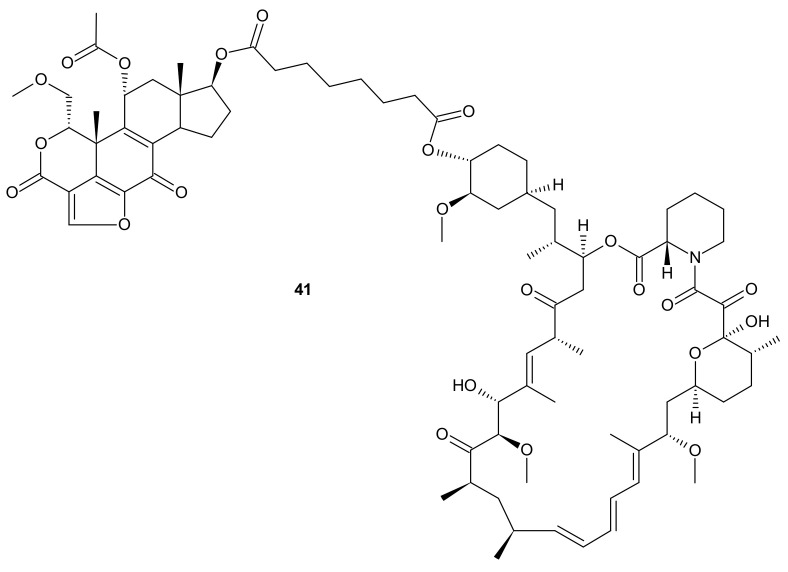
Structure of dual PI3K and mTOR inhibitor with excellent activity in vivo.

Kaloustian et al., conjugated 17-hydroxywortmannin (PI3K inhibitor) analog to rapamycin (mTOR inhibitor) through a diester linker which underwent hydrolysis in vivo and released two highly potent inhibitors of PI3K and mTOR. Conjugate **41** presented in Figure 21, showed profound activity in the mouse glioma model (U87MG). In addition, at the dose of 15 mg/kg, compound **41** (Figure 21) inhibited the growth of human colon adenocarcinoma (HT29) tumors, whereas an equivalent mixture of rapamycin and 17-hydroxywortmaninn was poorly tolerated. In the A498 renal tumor model, **41** (Figure 21) exhibited superior efficacy over rapamycin or 17-hydroxywortmaninn when administered as a single agent or in combination with bevacizumab [166].

Other examples of mTOR/PI3K dual inhibitors were extensively reviewed by Chen and Zhou [173].

**Figure 22:**
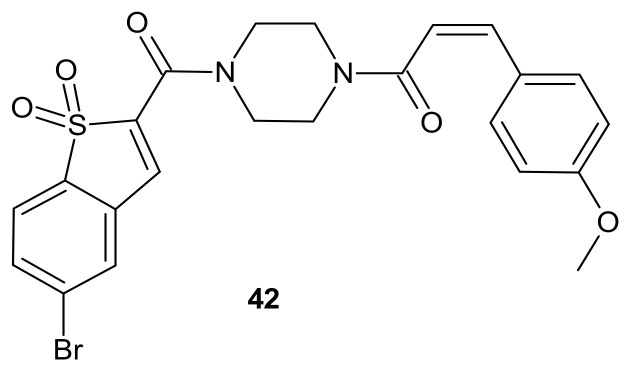
Curcumin-BTP hybrid—a STAT3 inhibitor with reactive oxygen species (ROS)-promoting activity.

The activation of a signal transducer and activator of transcription 3 (STAT3) signaling pathway is present in a wide range of solid cancers and drug-resistant cancers in humans. It is associated with a worse prognosis [174,175,176]. Therefore, inhibiting STAT3-mediated MDR1 gene expression is a possible treatment option for drug-resistant cancers [176]. Zhang et al., proposed an interesting idea for overcoming cancer cell resistance in solid tumors by designing a new series of curcumin-BTP hybrids as STAT3 inhibitors with reactive oxygen species (ROS)-promoting activity [177]. Curcumin and its analogs with improved anticancer potential and bioavailability inhibit numerous oncogenic processes, including those associated with the JAK2/STAT3 pathway or the induction of ROS production [178,179,180,181,182,183]. The benzo[b]thiophene 1,1-dioxide (BTP) moiety is a characteristic pharmacophore of many potent STAT3 inhibitors, markedly enhancing the ROS level and exhibiting antiproliferative activity in cancer cells [184,185]. Among obtained curcumin-BTP hybrids possessing drug-like properties and potent bioactivities in vitro and in vivo, **42** (Figure 22) exhibited the best antitumor activity and selectivity for MCF-7 and MCF-7/DOX cells, with significant STAT3 levels. Compound **42** (Figure 22) inhibited STAT3 activity, regulated the expression of STAT3 downstream genes Bcl-2, Bax and Cyclin D1, with little effect on p-Src or p-Erk, and also inhibited STAT3-mediated P-gp expression in MCF-7/DOX cells. In addition, **42** (Figure 22) was able to stimulate intracellular ROS production and accumulation. The aforementioned activity synergistically promoted cancer cell cycle arrest and apoptosis, which suggests that a combination of STAT3 inhibition with inducing high levels of ROS may be a valuable strategy to address resistance of cancer cells [177].

#### 3.2.6. Epigenetic Alterations

The modulation of epigenetic processes is nowadays considered an innovative and interesting therapeutic strategy because dysfunctional gene regulation is responsible for many human diseases [160] and emerging anticancer drug resistance [3]. Epigenetic modifications in gene expression cannot be explained by changes in DNA base sequences. They are related to covalent modifications to the cytosine residues of DNA, histone covalent chemical modifications such as acetylation, methylation, etc., known as chromatin remodeling, and noncoding RNAs [160]. Dysregulation of the dynamic status of histone acetylation plays an important role in the development of many malignancies [186]. The acetylation of lysine catalyzed by histone acetyltransferases (HATs) decreases the affinity of histones for DNA and opens the chromatin structure to the more relaxed euchromatin form. It allows for the binding of various transcription factors to DNA and activates many downstream gene transcription processes. Deacetylation of lysine performed by HDAC enzyme leads to chromatin compaction and gene silencing [187]. Therefore, HDAC inhibitors (HDACis) are epi-drugs that can reactivate signaling pathways silenced by deacetylation [160], which, in turn, leads to an increase in the expression of tumor suppressors or decreases the expression of genes involved in tumorigenesis [49]. HDACis exhibit multidirectional cellular effects, including the arrest of cell growth, cell cycle progression, and the induction of apoptosis. Moreover, through modification of both histone and non-histone substrates, they have the potential to disrupt multiple pathways of cancer resistance [188]. It was elucidated that HDAC inhibitors, co-administrated with other anticancer agents, enhanced their anticancer effects, which was the inspiration for the design of HDACIs-based hybrid drugs [50]. It must be underlined that the introduction of the pharmacophore for a second non-HDAC target would be impossible without a high degree of structural tolerance of the surface-binding cap, which enables the designing of multi-targeted molecules without compromising HDAC binding [50]. The number of multi-target HDACi hybrids reported in the literature is overwhelming [189,190]. The examples described below show an undeniable and significant role of these hybrids in overcoming anticancer drug resistance.

**Figure 23:**
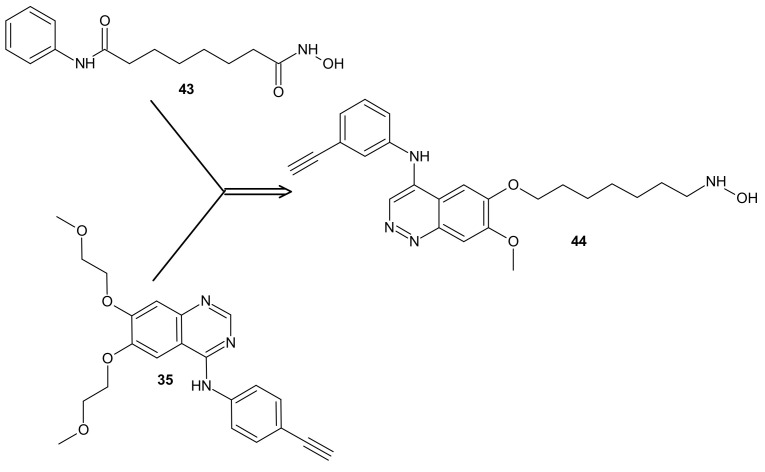
CUDC-101 **44** consists of a key fragment from the kinase inhibitor **35**, tethered by an appropriate linker to a zinc binding group from HDACi-SAHA **43**.

The design of RTKi/HDACi hybrids was proposed to overcome RTKi resistance, because combinations of HDACis and RTKis were shown to act synergistically [191,192,193]. Such a hybrid consists of a key fragment from the kinase inhibitor, tethered by the appropriate linker to a zinc binding group from HDACi, as shown in Figure 23 [194]. Many attempts were made to combine HDAC inhibitors and EGFR inhibitors in a single molecular entity [55,195,196,197].

Cai et al., combined HDAC inhibitor vorinostat (SAHA) **43** (Figure 23) with EGFR inhibitor erlotinib **35** (Figure 23), obtaining 7-(4-(3-ethynylphenylamino)-7-methoxyquinazolin-6-yloxy)-*N*-hydroxyheptanamide (CUDC-101) **44** (Figure 23) as a lead compound [198]. CUDC-101 **44** (Figure 23) is a multi-targeted entity that exhibited potent antiproliferative and proapoptotic activities against cancer cells in vitro and in drug-resistant tumor models in vivo. Cancer cells resistant to single-target EGFR inhibitors remain sensitive to **44** (Figure 23) [199]. It was elucidated that **44** (Figure 23) integrated HDAC and EGFR/HER2 pathway inhibition, blocked and inhibited MET- and AXL-mediated signaling, and reduced cancer cell migration [199,200]. Phase I study reported good tolerance of **44** (Figure 23) and showed some preliminary evidence of its antitumor activity [201].

Results of a multicenter phase I dose-escalation study in patients with locally advanced, intermediate-, or high-risk head and neck squamous cell cancer HNSCC also revealed good tolerance of **44** (Figure 23) in combination with cisplatin and radiation therapy. The maximum tolerated dose (MTD) of CUDC-101-based combination therapy was established at 275 mg/m^2^/dose [202].

Moreover, it was found that **44** (Figure 23) had potent antiproliferative and proapoptotic activities in anaplastic thyroid cancer (ATC) cell lines, with gene mutations in both the MAPK and the PI3K/AKT pathways [203]. ATC belongs to the most lethal human malignancies [204]. Treatment with **44** (Figure 23) reduced the expression of survivin, XIAP, N-cadherin, vimentin, β-catenin, and restored p21, as well as E-cadherin expression in ATC cells, which could also explain reduced tumor growth, metastasis, and prolonged survival observed in metastatic ATC mouse model in vivo [203].

In addition, **44** (Figure 23) exerted significantly stronger antiproliferative effects than arsenic trioxide (ATO) in Acute Promyelocytic Leukemia (APL) and ATO-resistant APL cell lines, whereas it has a negligible cytotoxic effect on non-cancerous cell lines, including normal CD34+ cells and BMSCs from APL patients. Mechanistic studies revealed that **44** (Figure 23) directly induced hyperacetylation of histone 3, which led to the activation of caspase 3 and the degradation of the promyelocytic leukemia-retinoic acid receptor α (PML-RARα) fusion protein, which subsequently facilitated the apoptosis of APL and ATO-resistant APL cells. Moreover, **44** (Figure 23) significantly repressed leukemia development in vivo in the NB4 xenograft mouse model compared with ATO. These results suggested that **44** (Figure 23) could be a potential candidate drug for APL, particularly for ATO-resistant APL [205,206].

**Figure 24:**
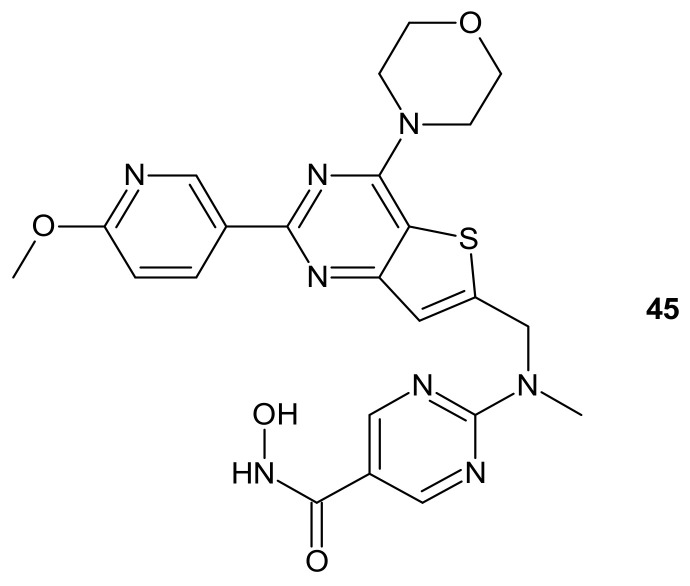
Structure of CUDC-907 (Fimepinostat).

CUDC-907 (Fimepinostat) **45** (Figure 24) is an orally available hybrid compound consisting of hydroxamic acid representing HDAC inhibitory functionality, tethered to a morpholinopyrimidine moiety—the core structure of the PI3K inhibitors. It is a nanomolar inhibitor of HDACs (I, II, Iv classes), as well as four I class kinases PI3K [207]. It (Figure 24) exhibited the potential to overcome drug resistance by simultaneous inhibition of PI3K activity and the disruption of cancer networks through epigenetic regulation of HDACs. It was elucidated that **45** (Figure 24) inhibited PI3K/AKT, JAK/STAT, and mitogen-activated protein kinase (MAPK) activation, as well as decreased C-MYC protein levels in solid tumor and hematological cell lines [208,209]. Moreover, the multidimensional modulation of dysregulated cancer pathways resulted in the significant antitumor activity of **45** (Figure 24) in many preclinical models of hematological malignancies and solid tumors [209,210,211,212,213,214], as well as clinical efficacy in relapsed/refractory lymphoma and multiple myeloma (MM) [215,216], MM, lymphoma, and advanced/relapsed solid tumors (newly diagnosed diffuse intrinsic pontine glioma (DIPG), recurrent medulloblastoma, or recurrent high-grade glioma (HGG)) in young adults and children [217,218].

**Figure 25:**
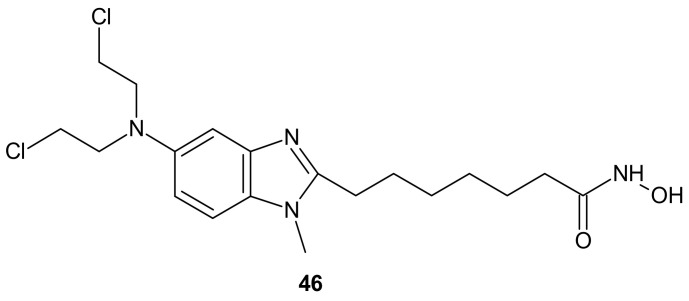
Structure of EDO-S101 (Tinostamustine).

An alkylating histonedeacetylase inhibiting molecule, EDO-S101 (Tinostamustine) **46** (Figure 25), obtained by the fusion of bendamustine with vorinostat, is the last example of a hybrid molecule described in our review. The rationale of its design was the synergy of HDAC-inhibitors and DNA-damaging agents relying on the HDACi-mediated chromatin relaxation that would make DNA more accessible to the damaging effect of bendamustine. EDO-S101 **46** (Figure 25) exhibited simultaneous alkylating activity and HDAC inhibition in vitro and in vivo [219]. Moreover, it showed significant activity against MM, leukemia, and B-cell lymphomas in preclinical models, with a toxicity profile similar to bendamustine [220]. It (Figure 25) was the only drug to exhibit activity as a single agent in the multidrug resistant, transplant model of relapsed/refractory MM [221]. This is in line with its outstanding synergistic cytotoxicity in combination with proteasome inhibitors against MM and a wide variety of B-cell neoplasms in vitro [222]. EDO-S101 **46** (Figure 25) is in early phase clinical development for a range of relapsed/refractory hematological malignancies, advanced solid tumors (small-cell lung cancer, soft tissue sarcoma, triplenegative breast cancer, ovarian cancer, endometrial cancer), and refractory, locally advanced or metastatic melanoma (with Nivolumab) [218]. In addition, it exhibits potent radio-sensitizing activity in aggressive and temozolomide-resistant glioblastoma tumor models, supporting ongoing clinical evaluation of this compound in combination with radiotherapy for the treatment of post-surgery glioblastoma patients [223]. It is well worth noting that the European Commission (EC) has adopted the recommendation of the European Medicines Agency (EMA) Committee for Orphan Medicinal Products to grant Orphan Drug Designation (ODD) to Tinostamustine **46** (Figure 25) for the treatment of T-cell prolymphocytic leukemia (T-PLL). The EC decision followed the FDA decision, which granted Tinostamustine ODD status for the treatment of T-PLL in March 2019 [224].

## 4. Conclusions

Despite enormous progress in the treatment of many malignant diseases that has been observed since the introduction of the first combination therapy, cancer cells are still able to develop resistance, leading to poor therapy outcomes. There is no doubt that hybrid drugs able to simultaneously inhibit more than one signaling pathway or target, without drug–drug interactions and with fewer side effects, play first fiddle in the field of discovery of novel and effective anticancer drugs.

In this paper, we attempted to highlight the possible role of hybrid drugs in overcoming cancer drug resistance. The abovementioned research results indicate the extraordinary potential of rationally designed multi-targeting hybrid drugs in resolving problems connected to chemotherapy failures.

## Data Availability

Not applicable.
